# Potential Molecular Mechanisms of Alzheimer’s Disease from Genetic Studies

**DOI:** 10.3390/biology12040602

**Published:** 2023-04-15

**Authors:** Martin Nwadiugwu, Hui Shen, Hong-Wen Deng

**Affiliations:** Tulane Center for Biomedical Informatics and Genomics, Deming Department of Medicine, Tulane University School of Medicine, Tulane University, New Orleans, LA 70112, USA

**Keywords:** Alzheimer’s disease, molecular mechanisms, single-cell transcriptomics, spatial genomics

## Abstract

**Simple Summary:**

We systematically reviewed genetic studies employing single-cell transcriptomics (scRNA-seq) or spatial genomics in Alzheimer’s disease (AD) research to highlight potential molecular mechanisms and explore what changed from previous findings. An overview of molecular imbalances in AD and differences in their mechanisms across sex, period of onset, age, and immunity are presented. Our study provides evidence for continuing research to identify common causes and possible solutions to ameliorate AD. scRNA-seq and spatial transcriptomics have the advantage of providing more in-depth results, which is vital for identifying crucial factors for AD-modifying investigations.

**Abstract:**

The devastating effects of Alzheimer’s disease (AD) are yet to be ameliorated due to the absence of curative treatment options. AD is an aging-related disease that affects cognition, and molecular imbalance is one of its hallmarks. There is a need to identify common causes of molecular imbalance in AD and their potential mechanisms for continuing research. A narrative synthesis of molecular mechanisms in AD from primary studies that employed single-cell sequencing (scRNA-seq) or spatial genomics was conducted using Embase and PubMed databases. We found that differences in molecular mechanisms in AD could be grouped into four key categories: sex-specific features, early-onset features, aging, and immune system pathways. The reported causes of molecular imbalance were alterations in bile acid (BA) synthesis, PITRM1, TREM2, olfactory mucosa (OM) cells, cholesterol catabolism, NFkB, double-strand break (DSB) neuronal damage, P65KD silencing, tau and APOE expression. What changed from previous findings in contrast to results obtained were explored to find potential factors for AD-modifying investigations.

## 1. Introduction

Alzheimer’s disease (AD) is one of the major causes of death in older people and the seventh leading cause of death in the United States [1]. It affects 1 in 9 Americans aged 65 and older and about 30 million people globally [2,3]. Despite the high economic cost and burden on caregivers, there are currently no known solutions that can ameliorate its devastating effects upon onset, as available medicinal interventions only provide symptomatic relief [4]. As a result, deaths from AD have continued to increase. About 145% increase in deaths from AD were estimated between 2000 and 2019, unlike deaths from heart disease which decreased by over 7% [3].

AD is a heterogenous aging disorder that affects cognition, and metabolic imbalance is one of its hallmarks. Therapeutic trials in AD are limited because they do not account for heterogeneity [5]. However, molecular genetic studies employing transcriptomics have continued to present novel findings while suggesting potential molecular mechanisms that may alter, delay, or halt AD pathogenesis. Single-cell transcriptomics (scRNA-seq) and spatial genomics are important methods because they can identify a heterogeneous population of cells and specific transcriptional information in a particular cell [6]. Both methods are very established techniques for exploring genotype–phenotype relationships with precise margins, and for mapping the location of occurrence of all gene activity within a single cell [7]. In spatial transcriptomics for instance, the mRNA is read in situ by FISH (fluorescence in situ hybridization), or microscopy, or other ex situ RNA sequencing to capture the RNA while retaining spatial information [8]. The relative advantage of spatial transcriptomics over scRNA-seq is the ability to clarify single-cell heterogeneity and characterize cell types while also preserving spatial information [8,9].

Methods that utilize both scRNA-seq and spatial mapping can capture the transcriptome topography in an unbiased fashion based on their tissue position before homogenization [10]. While poor subcellular location is still a limitation for spatial transcriptomics [10], it has been used to discover inflammatory microglia residing in specific brain regions that bear neurons with double strand breaks (DSB) in AD [11]. scRNA-seq on the other hand, has been employed to find gene expression in specific cells that other methods such as bulk RNA-seq may not be able to detect, due to the limitation of applying mixed cell populations [4,12]. Thus, scRNA-seq and spatial genomics can help in understanding the functional role of diverse cell types that are especially important for AD investigation.

Improvement in functional genomics discovery is one of the highlights of these two methods. Three unique marker genes (KIF5A, PAQR6, and SLC1A3) differentially expressed between AD and normal conditions were recently discovered with spatial transcriptomics [13]. Their differential expression showed significant differences in the white matter and cortical layer, as well as altered gene co-expression network patterns among various cell types in AD. Also, previously unknown molecular changes and cellular intercommunications that mark inflammatory responses about 100 uM diameter around amyloid plaques have been untangled using spatially resolved transcriptomics, leading to a differentiation in gene co-expression network patterns between early and late-onset AD [14]. Similarly, scRNA-seq have been employed to find differentially expressed genes (DEGs) in AD based on cell type, sex, and age [15]. Using scRNA-seq, Mathys et al. [15] reported that DEGs in early AD progression are cell type-specific and strongly expressed, unlike DEGs in late-onset AD that are common across cell types. scRNA-seq and spatial genomics, therefore, offers a robust blueprint for fine-grained cellular transcriptomics investigation.

This paper accesses the evidence base by systematically scoping the literature for genetic studies employing scRNA-seq or spatial genomics in AD research to identify potential molecular mechanisms. It provides synthesized information for continuing research to discover possible solutions to ameliorate AD. The overarching aim is to:

1. Identify common causes of molecular imbalance in AD;
2. Highlight potential molecular mechanisms.

The remaining sections of the paper will discuss the eligibility criteria, search strategy, evidence synthesis, and quality appraisal of the selected studies. This will be followed by an overview of molecular imbalances in AD and differences in their mechanisms across sex, period of onset, age, and immunity. Next, a narrative synthesis of potential molecular mechanisms of AD will be discussed with respect to the findings and what has changed in contrast to previous findings.

## 2. Materials and Methods

### 2.1. Inclusion and Exclusion Criteria

This study was registered in PROSPERO (CRD42022385085). Primary studies from any country that used human biological samples to study molecular mechanisms in AD and employed single-cell sequencing or spatial genomics were reviewed. Only original publications in English from peer-reviewed journals were included. The PCC framework suggested by the Joanna Briggs Institute (JBI) was used in defining the eligibility criteria seen below [16].

P—Population: Humans with Alzheimer’s disease

C—Concept: Molecular mechanisms

C—Context: Single-cell transcriptomics and/or spatial genomics. All articles reviewed reported molecular mechanisms in AD and applied genomics methods. Articles from grey literature and/or studies involving only non-human samples or other types of dementia or other neurogenerative diseases were excluded.

### 2.2. Search Strategy

The search was conducted in Embase and PubMed databases and covered the periods between 1960 and 2022. PubMed is one of the most popular biomedical and genomic databases accessed online from the National Center for Biotechnology Information (NCBI) platform. Similarly, Embase is another comprehensive pharmacological and biomedical bibliographic database hosted on Elsevier. All searches were conducted the same day, prior to 27 October 2022. The key categories searched were Alzheimer’s disease, single-cell transcriptomics and/or spatial genomics, and molecular mechanisms. These key search terms and their synonyms were identified using PROSPERO MeSH thesaurus qualifiers. Supplemental Table S1 shows the search items. The search queries can be seen in Supplemental Table S2. Each key term was individually searched. The logical “OR” operator was used to combine individual searches, and the intersection between them was performed using the “AND” operator.

The search strategy yielded a total of 93 hits. Thirty duplicates were removed after initial matching by PubMed ID and digital object identifier (DOI). Out of the 63 articles remaining, 46 were excluded for not fully meeting the selection criteria after screening by title and abstract. Eight articles were not included because they did not meet the eligibility criteria, leaving nine articles. The screening process is presented in Supplemental Figure S1 [17].

### 2.3. Charting the Data, Quality Appraisal, and Evidence Synthesis

To create a descriptive summary of the results with respect to the study objectives, data were extracted from the selected studies under these headings: author name and year, methodology adopted, study aims and sample type, key findings, category, and potential molecular mechanisms, as seen in Table 1.

The critical appraisal was performed using the Critical Appraisal Skills Programme (CASP) checklist with a modified set of questions appropriate for the study [18]. Table 2 shows the CASP checklist. It contained 11 questions that helped make sense of the study. The questions were based on the validity, results, and benefits of the selected study. The screening questions were answered quickly with a Yes (Y), No (N), Cannot tell (C), or Not applicable (N/A).

**Table 1:** Overview of the findings.

**Author Name and Year**	**Methodology Adopted**	**Study Aims and Sample Type**	**Key Findings**	**Category**	**Potential Molecular Mechanisms**
1. Varma et al. 2021 [19]	scRNA-Seq	Aims: the role of cholesterol catabolism in dementia Sample types: human blood serum	1. Low conc. of 7α-OHC and bile acid (BA) is linked with neuroimaging makers of dementia progression in males. 2. Pharmacological reduction of BA levels is associated with increased risk for vascular dementia in males but not in females.	1. Sex-specific 2. Early onset	1. BA signaling may be a novel target and BA synthesis could be a mediator of AD pathogenesis, mostly in males. 2. Early features of AD may be impacted by dysregulated cholesterol catabolism and BA synthesis.
2. Zalocusky et al. 2021 [20]	snRNA-seq	Aims: to find potential drivers of neuronal variability in AD. Sample types: human APOE-KI, ApoE3-KI, ApoE4-KIhomozygous mouse lines.	1. APOE pathway is related to DNA damage and repair, UPR, and immune response. Along with MCH-I, APOE may contribute to selective neurodegeneration.	1. Sex-specific 2.Immune system	1. Neuronal APOE expression might be a crucial factor driving within-neuron type variability. 2. Interaction between neuronal APOE and MHC-I may elicit AD tau pathology, present insults to microglia, and exhibit sex-dependent regulation.
3. Pérez et al. 2021 [21]	scRNA-Seq	Aims: to explore pathogenetic mechanisms of mitochondrialPITRM1 processing. Sample types: human organoid	1. Loss of PITRM1 function leads to AD-like pathological features. 2. PITRM1 deficient neurons show significantly low mitochondrial membrane potential and activates unfolded protein response. 3. Reduced neuronal loss, Aβ42/Aβ40 ratio, tau hyperphosphorylation, and mitochondrial clearance is improved via nicotinamide mononucleotide mitophagy stimulation.	1. Immune system	1. The mechanistic link between neurological proteinopathies and mitochondrial disorders may be explained by rare human PITRM1 mutations.
4. Welch et al. 2022 [11]	Bulk RNA-seq, snRNA-seq, spatial transcriptomics	Aims: to characterize DSB-bearing neurons from models of neurodegeneration. Sample types: postmortem human brain, CK-p25 mice	1. Neurons bearing DSBs enter late-stage DNA damage noticeable by NFkB–activated immune pathways and senescence. 2. Suppressed NFkB transcription factor in neurons reduces the spread and activation of microglia in both early and late AD, and rescues synapse loss. 3. NFkB regulates immune gene expression in DSB-bearing neurons, which secrete CCL2 and CXCL10 as primary signaling molecules to recruit and activate microglia.	1. Aging-related 2. Immune system	1. DSB accumulation with age may degrade neuronal chromatin integrity leading to immune activation that engages microglia. 2. DSB-induced immune gene expression and signaling may be linked with age decline in DNA repair. 3. Regulating immune activation and synaptic processes could be a primary role of neuronal NFkB. 4. Neurons with DSBs are possible neuroimmune relay hubs.
5. Fiock et al. 2020 [22]	scRNA-seq	Aims: to investigate how tau expression affects disease states by mapping its expression in the developing brain. Sample types: Human fetal sample, iPSC dorsal forebrain human cortical organoids	1. During neuronal maturation, tau expression increases in both the developing fetal brain and iPSC-derived organoids. 2. The maturation of migrating neuronal precursors increases tau mRNA expression, which begins in radial glia. 3. Low tau mRNA levels were found in deep white matter intermediate progenitors and subventricular zone radial glia.	1. Aging related	1. Regulatory mechanisms initiating tau gene transcription and translation in neurodegenerative tauopathies. 2. Tau mRNA expression may impact translational regulators of protein production and may precede onset of translation since it is turned on early in neuronal differentiation.
6. Lampinen et al. 2022 [12]	scRNA-seq	Aims: to evaluate OM differences between cognitively healthy and AD patients. Sample types: Human olfactory mucosa (OM) biopsies from healthy and late-onset AD patients.	1. AD OM cells had increased amyloid-beta secretion. 2. Multiple OM cells had altered signal transduction, RNA, protein metabolism, inflammatory and enriched immune system pathways. 3. From scRNA-seq data, eight genes were differentially expressed between the AD and control groups in entorhinal cortex, viz: HES1, BCYRN1, SERPINE1, MT-ND3, IFI27, MT-ND2, MAP1B, FTH1.	1. Sex-specific 2. Early onset	1. There may be a link between altered mitochondrial respiration and a reduction of ATP production in AD OM cells. 2. The OM and entorhinal cortex, which are vulnerable to early AD pathogenesis may exhibit disease specific changes. 3. There might be tissue-specific changes in low density lipoprotein receptor 1 (LRP1) in AD
7. Grubman et al. 2021 [23]	scRNA-seq, bulk RNA-seq	Aims: molecular and functional diversity of microglia cells in AD. Sample types: Paraffin-embedded human frontal cortex sections of postmortem AD and non-disease age-matched individuals. Transgenic mice overexpressing human FAD	1. Amyloid plaques without microglia (XO4-) show signatures of transcription linked with accelerated aging and increased intracellular postsynaptic material compared to those without (XO4+) 2. Aging microglia undergo transcriptional trajectory faster in AD, but upon plaque phagocytosis re-route to HIF1α regulon, resulting in increased Aβ phagocytosis.	1. Immune system	1. Phagocytic XO4+ microglia in AD may have detrimental or beneficial roles. 2. The targeted conversion between XO4− and XO4+ microglia and its key transcriptional network may be a potential intervention. 3. HIF1α potentially regulates synaptosome phagocytosis in vitro in primary human microglia.
8. Farmer et al. 2021 [24]	scRNA-Seq	Aims: investigate mechanism underlying changes in cerebral glucose metabolism in human expressing APOE4. Sample types: human DNA from blood samples, mice expressing human APOE.	1. Reduced oxygen consumption and increased plasma lactate drives low energy expenditure by redirecting flux through aerobic glycolysis in young female APOE4 carriers compared to non-carriers. 2. Participants with normal cognition with APOE genotypes show sex-specific APOE4-associated decrease in resting state respiratory quotient. 3. Increased glucose flux through aerobic glycolysis at the expense of oxidative phosphorylation and TCA cycle entry is linked with APOE4-expressing astrocytes.	1. Sex-specific 2. Early onset 3. Aging related 4. Immune system	1. APOE may have a role in immune-metabolism regulation and exhibit sex-specific roles in modulating cerebral and systemic glucose metabolism 2. Anaerobic metabolism may predict amyloid burden in later life since brain areas linked with aerobic glycolysis overlap with areas that accumulate amyloid β 3. Mechanism of energy expenditure and glucose metabolism in APOE2 carriers.
9. Okuzono et al. 2021 [4]	snRNA-Seq	Aims: to determine whether AD progression modifies TREM2 signaling. Sample types: human iPSC-derived hematopoietic progenitor cell	1. TREM2 activation was lower in AD microglia than in healthy subjects. 2. TREM2 activation is negatively correlated with AD. 3. TREM2 controls microglial functions to mediate regulation of AD. 4. In AD, activation of TREM2 may be associated with Th2-related pathways and microglial resistance. 5. Regardless of R47H (rare variant TREM2 loss-of-function) TREM2 signal is low in microglia in AD.	1. Immune system	1. TREM2 activation levels in the microglia of patients with MCI could indicate AD trajectory. 2. Activated monocytes may serve as a biomarker for microglial TREM2 AD activation since they correlate with TREM2 status in the microglia. 3. TREM2 activation in AD may lead to immune response, anti-apoptotic signaling, and cytoskeletal changes in the microglia.

The CASP checklist questions included:

1. Did the study address a clearly focused question?
2. Did the authors address the research aims and objectives?
3. Were the concept and context of the study measured and relevant?
4. Have the authors identified all necessary confounding factors?
5. Were the methods used sufficiently described in detail?
6. Were the results presented in a reproducible way?
7. Could the results have occurred by chance?
8. Can the result be applied to the population of interest?
9. Were all outcomes important to the population of interest?
10. Do the results of this study fit with other available evidence?
11. Are there implications of this study for practice?

As seen in Table 2, all papers had significant implications for the study objectives, described the population of interest and molecular mechanisms in AD, and applied either single-cell transcriptomics or spatial genomics methods (CASP checklist items 1, 2, 3, 5, 8, 11). It was unclear whether all confounding factors were identified in the 2nd, 3rd, 4th, and 5th studies because of the small sample size and/or lack of study limitations (CASP checklist items 4, 10). It could not be determined whether the 3rd and 6th studies were presented in a reproducible manner because of either a small cohort, experimental results that were not statistically significant, or reported sequencing results that are irreproducible, as seen especially in the 6th study (CASP checklist items 6, 7). It could not be determined whether the 1st, 5th, and 7th studies were in consensus with other available evidence because there was no comparable study. The 2nd and 6th studies, for instance, were unique because they were the first to report such findings. Not all outcomes from the 2nd, 4th, 7th, and 8th studies were equally important because some results were specific to non-human samples included in their respective studies (CASP checklist item 9).

## 3. Results

The results of the study are presented in Table 1. An overview of the findings showed that the potential molecular mechanisms in AD could be grouped into four key categories: sex-specific features, early-onset features, aging, and immune system features. Four studies [12,19,20,21] suggested potentially observable features of AD pathogenesis that may be sex-dependent. Three studies highlighted early-onset [12,19,21] and aging-related features [11,21,22], while six articles [4,11,20,21,23,24] highlighted immunity-related features in AD. In all, one study [21] covered all features, four studies [11,12,19,20] covered features in at least two categories, and the remaining studies [4,22,23,24] covered features in only one of the four broad categories.

### 3.1. Sex-Specific Molecular Mechanisms Underlying AD Pathogenesis

To understand the fundamental molecular mechanisms involved in AD pathogenesis, Varma et al. [19] found sex-specific differences in the altered gene expression of bile acid (BA) receptors in neurons and the association between bile acid sequestrants (BAS)—which lower bad cholesterol—and vascular dementia. Previous studies have shown a connection between reduced hippocampal volume and plasma cholic acid (a primary BA) [25]. Varma et al. [19] found that low levels of primary BAs, such as cholic and chenodeoxycholic acids, were linked with increased cerebral white matter lesions and atrophy in males compared to females. This led to speculation that disrupting primary BA synthesis could serve as a potential AD mediator, since BA synthesis is involved in pathologic changes before dementia onset in males, which points to BA alterations as a possible cause of molecular imbalance in AD. The study by Varma et al. [19] was one of the earliest to show sex-specific links between BA synthesis, accumulation of white matter lesions, amyloid deposition, and brain atrophy. Using scRNA-seq analysis, sex-specific progression of dementia was found to be associated with cholesterol and BA synthesis, and transcriptomic profiles of BA receptors such as peroxisome proliferator activated receptor alpha & gamma (PPARA & PPARG), retinoic acid receptor alpha (RARA), and retinoid x receptor alpha (RXRA) were differentially expressed in a sex-specific manner [19], which was consistent with previous results by Baloni et al. [26]. The scRNA-seq method allowed for multiple comparisons of whether the DEGs were sex-specific and/or from excitatory or inhibitory neurons. For instance, PPARA was found to be silenced while PPARG was only expressed in excitatory neurons in males. RARA, on the other hand, was highly expressed in excitatory neurons, while RXRA was mildly expressed in inhibitory neurons; neither RARA nor RXRA were significantly expressed in females. In excitatory neurons, the retinoid x receptor beta (RXRB) gene was silenced in females but highly expressed in males, while cholinergic receptor muscarinic 2 (CHRM2) was not significantly expressed in males but was downregulated in females [19]. It is important to note that PPARG and PPARA are involved in lipid and glucose metabolism [27,28], while CHRM2 has been linked with depressive disorder [29].

Nonetheless, alterations in neuronal apolipoprotein E (APOE) expression and olfactory mucosa (OM) cells also lead to sex-specific molecular differences in AD. In a female-only study conducted by Zalocusky et al. [20] to find drivers of selective neurodegeneration, the multiple major histocompatibility complex class 1 (MHC-I)—a gene which alerts the immune system to cells infected by viruses and beta-2-microglobulin genes—were found to be predicted by neuronal APOE expression. APOE is a major genetic risk factor and accounts for two-thirds of AD cases; the E4 allele of APOE is associated with higher AD risk [20]. In young females, carriers of the E4 allele of APOE had decreased daily energy expenditure; however, more studies are needed to establish sex-specific differences [21]. In OM cells, sex-specific differences were found in the study conducted by Lampinen et al. [12] to assess OM dissimilarities in AD patients. Unlike in males, female donors had significant elevated levels of low-density lipoprotein receptor-related protein 1 (LRP1), which is extensively expressed in the CNS and known to mediate endocytosis [12]. LRP1 has been touted to participate in regulating amyloid clearance, amyloid precursor protein trafficking, and blood–brain barrier permeability [30].

### 3.2. Early-Onset Molecular Mechanisms Underlying AD Pathogenesis

Dysregulation of BA synthesis and cholesterol catabolism may point to early features of dementia because the same pathway is entangled in neuropathological changes before onset of dementia [19]. This evaluation hypothesizes that targeting and modulating brain BA receptor–mediated signaling may increase the likelihood of identifying new dementia disease-modifying treatments [19].

Indeed, new treatment targets for AD prevention could be accelerated by understanding the mechanisms in young carriers of APOE4 who are yet to establish neuropathology. E4 carriers have been reported to display decreased cerebral glucose metabolism, which is a popular hallmark of early-onset AD [21]. The entorhinal cortex and OM are other possible distinctive targets for therapeutic approaches in AD because they are more likely to show disease-specific alterations and vulnerabilities in early AD pathogenesis [12]. The entorhinal cortex sits on the brain’s medial temporary lobe and is a hub for navigation, memory, and perception of time; while OM cells appear to be dysfunctional with increased amyloid β secretion in AD. Lampinen et al. [12], employing scRNA-seq identified eight DEGs (IFI27, MT-ND3, SERPINE1, MT-ND2, HES1, BCYRN1, MAP1B, and FTH1) in the entorhinal cortex between AD and normal conditions [12]. The entorhinal cortex and OM cells may serve as a critical investigation point to decipher risk for early AD, risk-related biomarkers, and therapeutic targets.

### 3.3. Aging-Related Molecular Mechanisms Underlying AD Pathogenesis

Another primary risk factor and important variable in understanding AD is aging [21]. DSBs accumulate with age, disrupting neuronal integrity and degrading chromatin to activate immune signaling and microglia [11]. It remains to be seen whether DSB-induced immune signaling is as a result of reduced DNA repair ability due to aging or by some other aging-related cellular functions that triggers immune signaling during neuronal distress [11]. However, in postmortem human brain, nuclear factor kappa B (NFkB) was identified as a major modulator of microglial activation via immune gene expression in neurons with DSBs [11]. NFkB’s primary neuronal function is to regulate synaptic activity, but it may also have a neuroprotective role. A potential molecular mechanism of NFkB is its aging-related regulation of CXCL10 (C-X-C motif chemokine ligand 10) and CCL2 (also known as MCP-1, monocyte chemoattractant protein 1), the primary signaling molecules from neurons bearing DSBs [11]. Recent studies have pointed to CXCL10 and CCL2 involvement in microglial activation and AD pathogenesis [11], and the merits of this requires investigation because neurons with DSBs are centers for neuroimmune communication and imbalance in CCL2 and CXCL10 axis may have damaging effects on cognition [11].

Nonetheless, as neurons age and mature in the developing brain, tau mRNA expression increases [22]. The onset of tau mRNA expression is observed very early in development and can be detected in radial glia and intermediate neuronal precursors [22]. Tau mRNA expression is understood to precede the onset of translation and is active during early neuronal differentiation [22].

### 3.4. Immune System Molecular Mechanisms Underlying AD Pathogenesis

The five important drivers of immune system molecular imbalance underlying AD from the selected studies were DSB neuronal damage, neuronal APOE expression, functional alterations of triggering receptor expressed on myeloid cells 2 (TREM2), absence of microglia in amyloid plaques, and defective mitochondria protease (PITRM1). Excitatory neurons with late-stage DSB damage were found to be linked with AD pathology and the immune response; these neurons are usually marked with antiviral immune activation in resemblance to a phenotype associated with virus-infected neurons and senescent cells [11]. In addition, these neurons disrupt microglial homeostasis and have two stages of active and amplified developmental immune signatures in AD that may play a functional role in neuroinflammation [11]. The suppression of DSB-bearing neuron immune signaling decreased microglial proliferation when P65KD—a gene that suppresses neuroimmune gene signatures—was knocked out [11]. This suggests that silencing P65KD may be a potential molecular mechanism in inhibiting neuroinflammation.

The differences in molecular characteristics of individual neurons under pathophysiological and normal conditions are driven by neuronal APOE expression [20]. A link between immunometabolism, the immune response, and neuronal APOE expression was established [21] after neuron-specific APOE knockout was found to redeem neuronal, hippocampal, and synaptic volume losses [20]. The discovery that variability in neuronal APOE expression plays a role in modulating the immune response and tracking AD progression is another additional piece of evidence [20]. It was hypothesized that upregulating neuronal APOE is the molecular switch that causes selective neurodegeneration, tau pathology, and unusual neuronal MHC-I levels due to abnormal synaptic homeostasis mediated by microglia and MHC-I (CD8+ T cells) [20]. In essence, increased MHC-I levels from stressed neurons are induced by neuronal APOE (the molecular switch), which could serve as a signal for phagocytosis. This provides an important point for future molecular studies with respect to selective neurodegeneration. Exploring the role of APOE in modulating immunometabolism [21] and investigating a combination of approaches in the APOE–MHC-I neuronal axis, as well as APOE-induced MHC-I overexpression, including mechanisms inhibiting APOE expression and MHC-I-induced tau pathology, could prove beneficial in AD research [20].

The functional alteration of TREM2—an innate immune system receptor expressed in microglia—[4] and the presence or absence of microglia in amyloid plaques [24] are drivers of molecular imbalance in AD. Okuzono et al. [4], studying the mechanisms of TREM2 activation in AD by investigating downstream expression changes in its antibody (Hyb87), found that unlike in healthy patients, TREM2 activation is decreased in microglia and may be linked with the type 2 immune response epitomized by pathways related to IL-4, T helper type 2 cells, and thymic stromal lymphopoietin. As AD progresses, the likelihood of TREM2 loss of function increases; this process is thought to support a protective role in AD because DEGs regulated by TREM2 would participate in the immune response, anti-apoptotic signaling, and changes in the microglial cytoskeleton upon TREM2 activation [4]. In addition, changes in the function and population of active monocytes were reported in AD patient blood samples, as peripheral immune cells showed elevated levels of activated monocytes [4]. Understanding the relationship between monocytes and TREM2 activation in AD could reveal potential molecular mechanisms and biomarkers with therapeutic benefits [4]. Nevertheless, amyloid plaques that do not contain microglia are considered to be on a path of accelerated aging, raising speculations that they follow another transcriptional trajectory for plaque and amyloid β-enhanced phagocytosis [24]. Therefore, investigations that target the shift from microglia-deficient amyloid plaques (XO4-) to amyloid plaques that contain microglia (XO4+) may clarify this supposition.

Defective mitochondria in AD are drivers of immune response reactions [23]. scRNA-seq data from cerebral organoids deficient in pitrilysin metallopeptidase 1 (PITRM1)—a mitochondrial matrix enzyme and protease—reportedly showed elevated TNF-α (an inflammatory cytokine) levels and changes in the astrocyte immune transcriptional signature [24]. PITRM1 participates in the processing and degrading of mitochondrial precursors and amyloid β [31]; it is an important enzyme because altering it results in incremental cognitive decline, psychotic episodes, and cerebellar ataxia [23]. Neurons deficient in PITRM1 show significant mitochondrial proteotoxicity and activation of unfolded protein response [23]. Research into rare human mutations of PITRM1 may provide an explanation on the mechanistic link between neurological proteinopathies and mitochondrial disorders.

### 3.5. What Changed from Previous Findings in Contrast to Results Obtained from scRNA-seq and Spatial Transcriptomics

Previous studies have used TREM2 activation tool, anti-TREM2 Ab, to observe cellular functions and visualize signals but not for TREM2 activation [4]. While these studies showed that TREM2 loss of function variant R47H was linked with increased neurofibrillary tangles and neuritic plaques in AD [32,33], Okuzono et al. [4] added that TREM2 loss was caused by proteases such as ADAM17, or ADAM10 and meprin β which inhibit TREM2-mediated phagocytosis. Employing scRNA-seq, Okuzono et al. [4] presented new information from gene set enrichment analysis (GSEA) that TREM2 loss of function is specific to AD but not significant in mild cognitive impairment (MCI) [4].

Furthermore, previous histological reports have suggested that OM cell alteration is an early symptom of AD; what changed was the cell type-specific functional alterations in OM that past reports did not reveal. Genes such as MT-ATP8 and MT2A, previously found in AD to be expressed in the frontal cortex and skin fibroblasts respectively, were also highly expressed in AD OM cells [12,34]. MT2A overexpression in HEK cells was reported to show mitochondrial dysfunction in a manner hypothesized to similarly occur in AD OM cells [35,36].

With respect to APOE expression, results show that it is more elevated in E4 than in E3 astrocytes, and scRNA-seq analysis by Farmer et al. [21] have identified more astrocyte-specific DEGs (526) between E4 and E3, unlike previous bulk RNA-seq results that revealed non-astrocyte-specific DEGs. Additionally, recent studies have observed sex-specific but not aging-related decline in the relationship between resting energy expenditure and E4, compared to a negative aging-related correlation reported in previous studies. It has been evidenced that neuronal rather than astrocytic APOE expression increases the phosphorylation of tau, nonetheless, results from a scRNA-seq study by Zalocusky et al. [20] found neuronal MHC-I as the driver through which neuronal APOE expression acts to elicit tau pathology. Moreover, previous studies have found immature brain regions to exhibit low tau mRNA expression, which increases with neuronal growth and is active during early neuronal differentiation [37]. Fiock et al. [22] agreed with this finding, drawing from similar observations from their study that utilized human cortical organoids as a high-fidelity model for developmental expression of tau instead of rodents [37,38]. Indeed, by querying marker genes against cell type-specific markers, scRNA-seq analysis from these studies identified important molecular relationships and DEGs from diverse cell classes.

A list of 2031 new genes previously unconnected with amyloid plaques containing microglia and highly enriched in AD were found by Grubman et al. [24] after applying scRNA-seq for specific profiling and sequencing to a greater depth. scRNA-seq uncovered more overlapping AD and HIFLA-related DEGs (536) associated with amyloid plaques containing microglia than bulk RNA-seq analysis (344), proving that scRNA-seq is better for identifying more specific information from heterogeneous cell populations. Some of the overlapping upregulated genes include TREM2, APOE, CST7, and TYROBP [23]. In addition, scRNA-seq have allowed for demonstrable cell type-specific loss of PITRM1 function in brain cells [23]. Apart from progenitor cells, PITRM1 was observed to be involved in pathways connected with mitochondrial functions in many brain cell types. According to Perez et al. [23], the loss of PITRM1 function in the immune system pathway in AD leads to dissimilar astrocyte signatures; however, unlike findings from other studies [39], microglia-relevant pathways were not significant.

Despite the limitations of spatial transcriptomics, it is continuing to prove its importance. A previous study [40] showed NFkB as a treatment target for AD, because when silenced by p65 knockdown it reduced activated microglial gene expression and morphology, however, recent spatial transcriptomics analysis by Welch et al. [11] further revealed that NFkB knockdown played a similar pivotal role in DSB-bearing neurons. DSB-bearing neurons are indicated by the presence of λH2A.X, which is phosphorylated by ataxia-telangiectasia mutated kinase [11]. Using spatial transcriptomics, Welch et al. [11] captured areas of the brain (cortex, dentate gyrus, and hippocampal CA3, CA2, and CA1 areas) with λH2A.X signals and differentially compared them using GSEA to find microglial signatures.

## 4. Discussion

Identifying molecular level mechanisms in AD is limited due to the lack of data and difficulty in obtaining samples from the brains of patients [41]. However, genes found from genetic studies that regulate molecular imbalance can be used as diagnostic markers. Sex-specific associated risk and progression have been observed in AD. In males for example, reduced neuronal gene expression of BA receptors and BA levels are associated with higher AD risk, while increased risk of AD is linked with females carrying APOE4 and those with elevated levels of LRP1 in OM fibroblast cells [12,19,20]. Investigating whether impaired BA synthesis is a mediator of AD pathogenesis and whether BA signaling, and dysregulation of cholesterol catabolism is a novel target that could impact dementia is beneficial because altered BAs and their mRNA receptors are differentially expressed in AD and normal conditions [19]. LRP1 shows sex-specific differences; it plays a role in amyloid β clearance from the brain and is significantly elevated in OM cells of females compared to males [42]. It is also differentially expressed in OM fibroblast cells and brain endothelial cells in AD [42]. While sex-specific differences have been reported in AD pathogenesis, understanding whether these sex-related differences also show sex-dependent molecular regulation would add to the current evidence. 

In early-onset AD, the key to better management is early detection, which is quite challenging due to misdiagnosis [43]; therefore, identifying new AD-modifying treatment options is vital. One suggestion is the evaluation of dysregulated BA synthesis and the cholesterol catabolism pathway, which appears to be wrapped in neuropathological changes before early onset of dementia [19]. Another plausible target is understanding the molecular mechanisms of aerobic and anaerobic glycolysis in APOE4 carriers, which were found to exhibit a pro-glycolytic shift in young women [21]. This is important because areas of the brain that accumulate amyloid β overlap with areas of the brain linked with aerobic glycolysis; as a result, studies on anaerobic metabolism are hypothesized as a predictor of amyloid burden in later life [24]. Moreover, disease alterations in the entorhinal cortex and OM are both vulnerable to early AD pathogenesis [12]. OM cells in AD show increased amyloid β secretion, altered RNA, signal transduction, and protein metabolism, while genes in the entorhinal cortex (eight genes) have been observed to be differentially expressed between AD and normal conditions [12]. Hence, research on disease-specific changes in OM cells and the entorhinal cortex may uncover new early-onset risk-related biomarkers. 

The accumulation of deficits and distinct changes in gene expression and tissue regulation have been reported in several aging-related studies [11,21,22,44]. Deficits such as the accumulation of DSBs in neurons disrupt neuroimmune communication and cause late-stage DNA damage [11]. DSBs are regulated by NFkB, which is suggested to be neuroprotective because it plays a role in modulating CXCL10 and CCL2 imbalance and in microglial activation [11]. As amyloid plaques containing microglia age in AD, they undergo a faster transcriptional trajectory leading to increased intracellular postsynaptic material [24]. Understanding the aging-related cellular functions of NFkB in relation to DSBs could unpack its primary neuronal role in immune activation, synaptic processing, synaptosome phagocytosis, and DSB-induced immune signaling. As the brain develops and ages, tau gene expression increases and mediates neurotoxicity in AD. Knowledge of regulatory mechanisms initiating tau gene transcription and translation in neurodegenerative tauopathies may prove helpful for tau therapies that are increasingly being investigated due to lack of success in therapies targeting amyloid β [22].

The loss of TREM2 and PITRM1 function may give rise to AD-like pathological features [4,23]. TREM2 activation is a driver of the immune response and negatively correlates with AD [4]. TREM2 controls microglial function; in AD, it correlates with activated monocytes and low activation in microglia, where it is associated with microglial resistance, anti-apoptotic signaling, and cytoskeletal changes [4]. However, some essential questions for exploration include: (1) can TREM2 activation be maintained in the microglia of AD patients with mild dementia, and (2) can activated monocytes serve as a biomarker for microglial TREM2 activation in AD since they correlate with TREM2 status? Evaluating the molecular basis and mechanisms of these questions may be beneficial to avoid TREM2 loss of function as AD progresses. 

It is important to consider the hallmarks of underlying immune system molecular mechanisms in AD pathogenesis. The APOE pathway is related to DNA damage and repair; together with MCH-I, APOE expression is a crucial factor driving immune response and variability within individual neurons [20]. Likewise, accumulation of DSBs with age promotes DNA damage by degrading neuronal chromatin integrity, leading to immune activation that engages microglia [11]. Since the APOE pathway, MHC-I, and DSB accumulation promotes immune response and an unstable genome, investigating whether there is a mechanistic connection between them will be useful. Mechanistic linkages may reveal hallmarks such as neuroinflammation and genome fragility which may be of importance in AD research [11].

## 5. Conclusions

Taken together, the results across all four categories show that the reported causes of molecular imbalance are alterations in BA synthesis, loss of PITRM1 and TREM2 function, disruption of OM cells, dysregulated cholesterol catabolism, DSB neuronal damage, NFkB and P65KD silencing, tau expression, and neuronal APOE expression. Potential molecular investigations may evaluate the impact of altering BA signaling and synthesis, sex-specific role of LRP1 regulation, AD-specific changes in OM cells and the entorhinal cortex, regulatory mechanisms initiating tau gene transcription, microglial TREM2 activation, and PITRM1 mechanistic links between mitochondrial disorders and neurological proteinopathies. Evaluating these molecular imbalances could reveal crucial factors for AD-modifying investigations and targeted treatments.

## Figures and Tables

**Table 2:** CASP checklist.

S/N	Author, Year	CASP Checklist Items										
		1	2	3	4	5	6	7	8	9	10	11
1	Varma et al., 2021 [19]	Y	Y	Y	Y	Y	Y	C	Y	Y	C	Y
2	Zalocusky et al., 2021 [20]	Y	Y	Y	C	Y	Y	N	Y	N	N/A	Y
3	Pérez et al., 2021 [21]	Y	Y	Y	C	Y	C	N	Y	Y	Y	Y
4	Welch et al., 2022 [11]	Y	Y	Y	C	Y	Y	N	Y	N	Y	Y
5	Fiock et al., 2020 [22]	Y	Y	Y	Y	Y	Y	C	Y	Y	C	Y
6	Lampinen et al., 2022 [12]	Y	Y	Y	Y	Y	C	N	Y	Y	N/A	Y
7	Grubman et al., 2021 [23]	Y	Y	Y	N	Y	Y	N	Y	N	C	Y
8	Farmer et al., 2021 [24]	Y	Y	Y	Y	Y	Y	N	Y	N	Y	Y
9	Okuzono et al., 2021 [4]	Y	Y	Y	C	Y	Y	C	Y	Y	Y	Y

Green: studies that met the CASP checklist question, Orange: studies that did not meet specific CASP checklist question, Yellow: CASP checklist questions that cannot be answered, No color: CASP checklist question that were not applicable.

## Data Availability

No new data were created in this study. Data sharing is not applicable to this article.

## Supplementary Materials

The following supporting information can be downloaded at: https://www.mdpi.com/article/10.3390/biology12040602/s1, Supplemental Figure S1: PRISMA flow diagram; Supplemental Table S1: Search strategy; Supplemental Table S2: Search queries.
