# Supplementary material for: Potential Molecular Mechanisms of Alzheimer’s Disease from Genetic Studies
Source: Biology (Basel). 2023 Apr 15;12(4):602. doi: 10.3390/biology12040602 (PMC10136191; doi:10.3390/biology12040602)
Supplement: Supplementary file 1 [file biology-12-00602-s001.zip › biology-2311405-Supplementary.pdf]

Supplemental Table S1: Search strategy

	Term 2 (Single cell transcriptomics, spatial genomics)	Term 3 (Molecular mechanisms)
"AD" or "Alzheimer Dementia" or "Alzheimer Dementias" or "Alzheimer Disease Early Onset" or "Alzheimer Disease, Familial (FAD)" or "Alzheimer Disease Late Onset" or "Alzheimer Diseases" or "Alzheimer Diseases Familial (FAD)" or "Alzheimer Sclerosis" or "Alzheimer Syndrome" or "Alzheimer Type Dementia" or "Alzheimer Type Dementia (ATD)" or "Alzheimer Type Senile Dementia" or "Alzheimer's Disease" or "Alzheimer's Disease Focal Onset" or "Alzheimer's Diseases" or "Alzheimers Diseases" or "Alzheimer-Type Dementia (ATD)" or "Dementia Alzheimer" or "Dementia Alzheimer Type" or "Dementia Alzheimer-Type (ATD)" or" Dementias Alzheimer" or "Early Onset Alzheimer Disease" or "Familial Alzheimer Disease (FAD)" or "Familial Alzheimer Diseases (FAD)" or "Focal Onset Alzheimer's Disease" or "Late Onset Alzheimer Disease" or "Presenile Alzheimer Dementia" or "Sclerosis Alzheimer" or "Senile Dementia Alzheimer Type"	"Single cell sequencing" or "scrnaseq" or "single-cell RNA-seq" or "scRNA-seq" or "single cell RNA-seq" or "single-cell transcriptomics" or "single-nucleus RNA-sequencing" or "snRNA-seq" or "spatial transcriptomics" or "spatial genomics"	"Molecular mechanisms" or molecular mechanism* or cellular mechanism* or biology mechanism*

Supplemental Table S2: Search queries

**Database**	**Source**
PubMed	PubMed: https://pubmed.ncbi.nlm.nih.gov/advanced/<br><br>Search Strings:<br><br>(("AD" or "Alzheimer Dementia" or "Alzheimer Dementias" or "Alzheimer Disease Early Onset" or "Alzheimer Disease, Familial (FAD)" or "Alzheimer Disease Late Onset" or "Alzheimer Diseases" or "Alzheimer Diseases Familial (FAD)" or "Alzheimer Sclerosis" or "Alzheimer Syndrome" or "Alzheimer Type Dementia" or "Alzheimer Type Dementia (ATD)" or "Alzheimer Type Senile Dementia" or "Alzheimer's Disease" or "Alzheimer's Disease Focal Onset" or "Alzheimer's Diseases" or "Alzheimers Diseases" or "Alzheimer-Type Dementia (ATD)" or "Dementia Alzheimer" or "Dementia Alzheimer Type" or "Dementia Alzheimer-Type (ATD)" or" Dementias Alzheimer" or "Early Onset Alzheimer Disease" or "Familial Alzheimer Disease (FAD)" or "Familial Alzheimer Diseases (FAD)" or "Focal Onset Alzheimer's Disease" or "Late Onset Alzheimer Disease" or "Presenile Alzheimer Dementia" or "Sclerosis Alzheimer" or "Senile Dementia Alzheimer Type") AND ("Single cell sequencing" or "scrnaseq" or "single-cell RNA-seq" or "scRNA-seq" or "single cell RNA-seq" or "single-cell transcriptomics" or "single-nucleus RNA-sequencing" or "snRNA-seq" or "spatial transcriptomics" or "spatial genomics")) AND ("molecular mechanisms" or molecular mechanism* or cellular mechanism* or biology mechanism*)
Embase	Embase: https://www.embase.com/#advancedSearch/default<br><br>Search Strings:<br><br>((('ad' OR 'alzheimer dementia'/exp OR 'alzheimer dementia' OR 'alzheimer dementias' OR 'alzheimer disease early onset' OR 'alzheimer disease, familial (fad)' OR 'alzheimer disease late onset' OR 'alzheimer diseases' OR 'alzheimer diseases familial (fad)' OR 'alzheimer sclerosis'/exp OR 'alzheimer sclerosis' OR 'alzheimer syndrome'/exp OR 'alzheimer syndrome' OR 'alzheimer type dementia' OR 'alzheimer type dementia (atd)' OR 'alzheimer type senile dementia' OR alzheimers* OR 'alzheimers disease focal onset' OR 'alzheimers diseases' OR 'alzheimer-type dementia (atd)' OR 'dementia alzheimer'/exp OR 'dementia alzheimer' OR 'dementia alzheimer type' OR 'dementia alzheimer-type (atd)' OR 'dementias alzheimer' OR 'early onset alzheimer disease'/exp OR 'early onset alzheimer disease' OR 'familial alzheimer disease (fad)' OR 'familial alzheimer diseases (fad)' OR 'focal onset alzheimers disease' OR 'late onset alzheimer disease'/exp OR 'late onset alzheimer disease' OR 'presenile alzheimer dementia' OR 'sclerosis alzheimer' OR 'senile dementia alzheimer type') AND [<1966-2022]/py) AND (('single cell sequencing'/exp OR 'single cell sequencing' OR 'scrnaseq'/exp OR 'scrnaseq' OR 'single-cell rna-seq'/exp OR 'single-cell rna-seq' OR 'scrna-seq'/exp OR 'scrna-seq' OR 'single cell rna-seq'/exp OR 'single cell rna-seq' OR 'single-cell transcriptomics' OR 'single-nucleus rna-sequencing' OR 'snrna-seq' OR 'spatial transcriptomics'/exp OR 'spatial transcriptomics' OR 'spatial genomics') AND [<1966-2022]/py) AND (((('molecular mechanisms' OR molecular) AND mechanism* OR cellular) AND mechanism* OR 'biology'/exp OR biology) AND mechanism* AND [<1966-2022]/py)) AND 'alzheimer disease'/dm AND 'human'/de

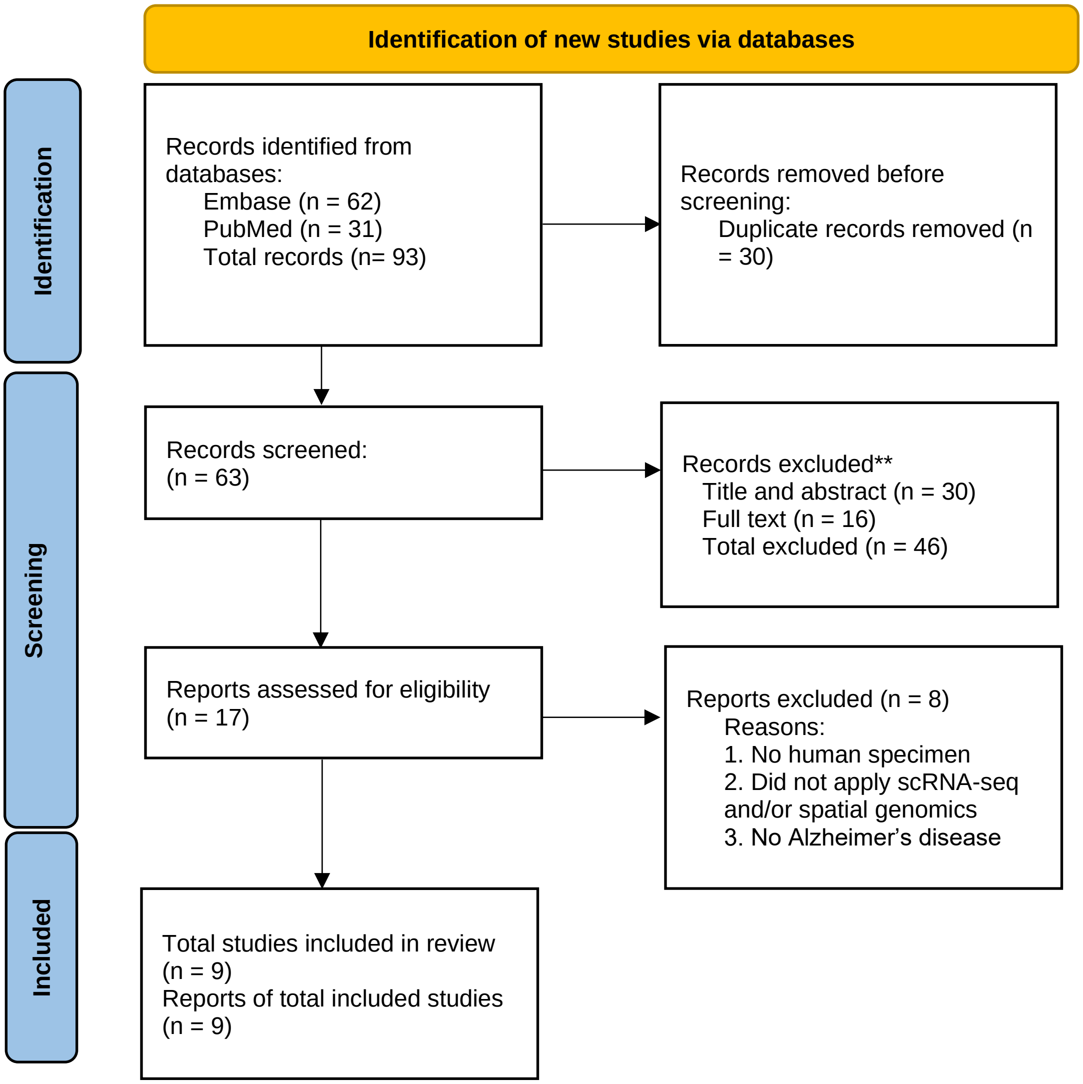

Supplemental Figure S1: PRISMA flow diagram.
